# Hemoglobin concentrations and adverse birth outcomes in South Asian pregnant women: findings from a prospective Maternal and Neonatal Health Registry

**DOI:** 10.1186/s12978-020-01006-6

**Published:** 2020-11-30

**Authors:** Sumera Aziz Ali, Shiyam Sunder Tikmani, Sarah Saleem, Archana B. Patel, Patricia L. Hibberd, Shivaprasad S. Goudar, Sangappa Dhaded, Richard J. Derman, Janet L. Moore, Elizabeth M. McClure, Robert L. Goldenberg

**Affiliations:** 1 Department of Community Health Sciences, Aga Khan University, Stadium Road, Karachi, 74800 Pakistan; 2 Lata Medical Research Foundation, Nagpur, India; 3 Boston University, Boston, MA USA; 4 KLE Academy of Higher Education and Research’s J N Medical College, Belagavi, India; 5 Thomas Jefferson University, Philadelphia, PA USA; 6 RTI International, Research Triangle Park, Durham, NC USA; 7 Department of Obstetrics and Gynecology, Columbia University, New York, NY USA

**Keywords:** Hemoglobin concetrations, Anemia, South Asia, India, Pakistan, Pregnancy outcome, Stillbirth, Neonatal mortality, Global network

## Abstract

**Background:**

While the relationship between hemoglobin (Hb) concentrations and pregnancy outcomes has been studied often, most reports have focused on a specific Hb cutoff used to define anemia. Fewer studies have evaluated pregnancy outcomes across the entire range of Hb values. Moreover, to date, most studies of the relationship of Hb concentrations to pregnancy outcomes have been done in high-income countries. Thus, we have sought to determine the relationship between the range of maternal Hb concentrations and adverse birth outcomes among South Asian pregnant women.

**Methods:**

For this study, we used data collected from two South Asian countries (Pakistan – Sindh Province and two sites in India - Belagavi and Nagpur) in a prospective maternal and newborn health registry study. To assess the association between Hb concentrations and various maternal and fetal outcomes, we classified the Hb concentrations into seven categories. Regression analyses adjusting for multiple potential confounders were performed to assess adverse pregnancy outcomes across the range of Hb concentrations.

**Findings:**

Between January 2012 and December 2018, 130,888 pregnant women were enrolled in the South Asian sites had a Hb measurement available, delivered and were included in the analyses. Overall, the mean Hb concentration of pregnant women from the sites was 9.9 g/dL, 10.0 g/dL in the Indian sites and 9.5 g/dL in the Pakistan site. Hb concentrations < 7 g/dL were observed in 6.9% of the pregnant Pakistani women and 0.2% of the Indian women. In both the Pakistani and Indian sites, women with higher parity and women with no formal education had lower Hb concentrations. In the Pakistani site, women > 35 years of age, women with ≥4 children and those who enrolled in the third trimester were more likely to have Hb concentrations of < 7 g/dL but these associations were not found for the Indian sites. When adjusting for potential confounders, for both India and Pakistan, lower Hb concentrations were associated with stillbirth, preterm birth, lower mean birthweight, and increased risk of low birthweight. In the Pakistani site, there was evidence of a U-shaped relationship between Hb concentrations and low birth weight, and neonatal mortality, and in India with hypertensive disease.

**Interpretation:**

This study documented the relationship between maternal Hb concentrations and adverse pregnancy outcomes in women from the Pakistani and Indian sites across the range of Hb values. Both low and high Hb concentrations were associated with risk of at least some adverse outcomes. Hence, both low and high values of Hb should be considered risk factors for the mother and fetus.

## Background

Anemia remains a significant health problem globally, accounting for more than 60,000 maternal deaths and 3.4% of global disability-adjusted life years in women aged 15–49 years [1]. According to the World Health Organization (WHO), globally, 528.7 million (29.4%) women of reproductive age are anemic with a hemoglobin (Hb) concentration of < 11 g/dL [2]. Of these women, 20.2 million are defined as severely anemic with a Hb concentration of < 7 g/dL [2]. Rates of anemia are highest in low-resource countries, especially in central and west Africa where 48% of reproductive-age women and 56% of all pregnant women are reported to be anemic and in South Asia, where 47% of all reproductive-age women and 52% of pregnant women are reported to be anemic [2].

Multiple adverse maternal and neonatal outcomes have been attributed to anemia [3]. These outcomes vary according to the severity of anemia [4]. Reported maternal and perinatal outcomes among severely anemic women include premature rupture of membranes, preterm births (PTB), hypertensive diseases of pregnancy, puerperal pyrexia, fetal distress, small for gestational age, stillbirths, neonatal and maternal deaths [5]. Findings from systematic reviews and meta-analyses have also suggested that in low-income countries, 25% of low-birth weight (LBW), 44% of PTB, and 21% of perinatal mortality are attributable to anemia [6]. One review observed a relatively higher anemia-attributable proportion of LBW in Pakistan and Bangladesh compared to Ghana and India [6]. Similarly, the highest anemia-attributable proportion of PTB was observed in Pakistan (54%) followed by India (27%) and Iran (18%) [6]. Further, studies have also revealed that women with low Hb concentrations during pregnancy are at higher risk of antepartum and postpartum hemorrhage, obstructed labor, and cesarean-section delivery when compared to the women with normal hemoglobin concentrations [7–10].

At the other end of the spectrum, several older studies have shown that elevated Hb concentrations during pregnancy are also associated with increased risk of adverse birth outcomes, including PTB, LBW, fetal death and intrauterine growth retardation [11–14]. However, the findings have not been consistent [12, 15]. Moreover, this potential U-shaped relationship, with higher risks of adverse birth outcomes at both extremes of Hb concentrations have been assessed primarily in more developed countries such as the U.S., Sweden, and Iran [16–18]. Thus, very few studies have described the relationship between high Hb concentrations and birth outcomes in low-and middle-income countries (LMIC) and particularly in women from South Asia [11, 12]. Moreover, the relationship between higher concentrations of Hb with adverse outcomes, such as antepartum and postpartum hemorrhage, obstructed labor, and cesarean delivery is not explored in the literature.

The objective of this study was to assess the associations across the range of maternal Hb concentrations and adverse birth outcomes in South Asian pregnant women as well as evaluating factors related to Hb concentrations by country. Differences between the Pakistani and Indian sites in the relationship of Hb concentration and pregnancy outcomes were explored. An understanding of these outcomes and the association of Hb concentrations with adverse pregnancy outcomes is essential to inform policies to improve maternal and fetal/neonatal outcomes.

## Methods

The Global Network’s Maternal Newborn Health Registry (MNHR) is a multi-site, prospective, ongoing, active surveillance system to track pregnancies and births in defined geographic communities (clusters), each with approximately 300 to 500 deliveries per year. The MNHR is funded by the *Eunice Kennedy Shriver* National Institute of Child Health and Human Development (NICHD) through grants to the NICHD Global Network for Women’s and Children’s Health (ClinicalTrials.gov Identifier: NCT01073475). The aim of the MNHR is to document birth outcomes in defined geographical areas and provide population-based rates of stillbirth, neonatal and maternal deaths, and other adverse outcomes. The details of the MNHR are described elsewhere [19].

**Fig. 1:**
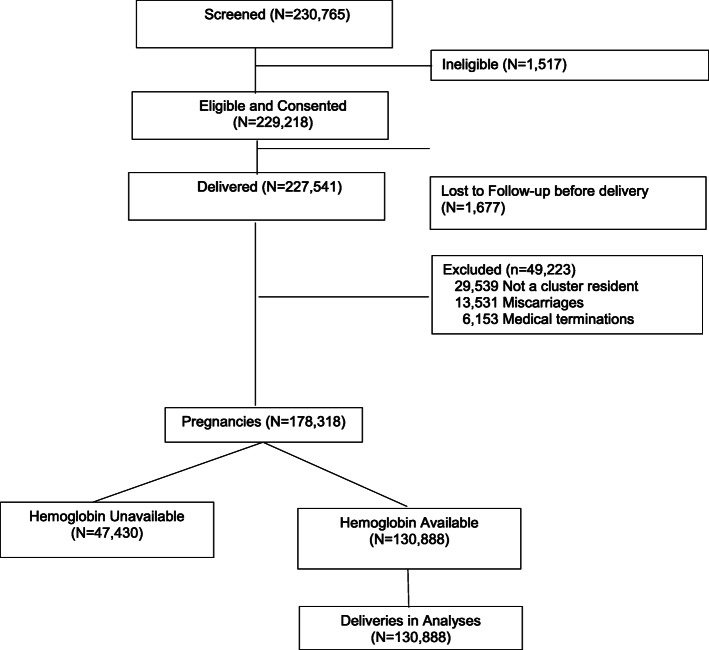
Diagram of Maternal Newborn Health study enrollment in India and Pakistan Global Network sites, 2012–2018

For this study, we used data collected from the three South Asian sites (Pakistan – Sindh Province and two sites in central India - Belagavi and Nagpur). For purpose of analyses, we grouped the two Indian sites, which had similar demographics, and analyzed the Pakistan site separately. Since the MNHR began to collect maternal Hb concentrations in 2012, we included data of women who were enrolled between January 2012 and December 2018. However, the Pakistani site started routinely collecting Hb data in 2014. Those pregnant women who provided consent and had Hb measurements available at the enrollment visit were included. Women who delivered before 20 weeks, had a medical termination of pregnancy, were not residents of the study cluster or who had incomplete outcome data were excluded from this analysis (Fig. 1).

Gestational age was based on the best data available, usually the last menstrual period (LMP), although in the later years of the study, ultrasound was more widely used for gestational age determination. PTB was defined as births < 37 weeks’ gestational age using a project specific algorithm. The gestational age of the first prenatal visit was used as a surrogate for the gestational age of the Hb determination, because Hb was generally measured at the first prenatal visit and we did not collect the specific gestational age Hb measurement separately. If more than one Hb measurement was recorded, only the first was used in this analysis. The inter-delivery interval was calculated by subtracting the date of the last delivery from the date of delivery of the current pregnancy and converting that time into months.

To assess the association between Hb concentrations and various maternal outcomes, (maternal mortality, antepartum hemorrhage, postpartum hemorrhage, hypertensive disease of pregnancy/pre-eclampsia/eclampsia, obstructed labor and cesarean delivery) and fetal/neonatal outcomes (PTB, LBW, stillbirth and neonatal mortality), we classified the Hb concentrations into 7 categories, < 7.0 g/dL, 7.0–7.9 g/dL, 8.0–8.9 g/dL, 9.0–9.9 g/dL, 10.0–10.9 g/dL, 11.0–12.9 g/dL and ≥ 13.0 g/dL. Values of 11.0–12.9 g/dL are generally considered normal^17^ and were used as the reference group for this study to assess the relationship of outcomes with other Hb categories.

Data were entered and edited at each study site prior to secure transmission to the central data center (RTI International) where further edits and statistical analyses were performed. We produced descriptive statistics stratified by country to examine the different concentrations of Hb for characteristics of pregnant women and used Cochran-Mantel-Haenszel tests stratified by study cluster to assess differences in hemoglobin concentrations among these characteristics. Generalized linear models adjusting for multiple potential maternal confounders and using generalized estimating equations to account for the correlation of outcomes within cluster were performed to assess the risk of adverse pregnancy outcomes across the range of Hb concentrations compared to the reference group separately for the Indian and Pakistani sites. In these models, we also adjusted for the gestational age at enrollment, as there may be confounding associated with gestational age of Hb measurement and outcomes. We then performed locally weighted scatterplot smoothing (LOESS), a non-parametric method for fitting a smooth curve to data points to picture the relationship between maternal Hb concentrations and four birth outcomes (birth weight, stillbirth, 7-day neonatal mortality and 28-day neonatal mortality).

This study was reviewed and approved by all sites’ ethics review committees (Aga Khan University, Karachi, Pakistan; KLE Academy of Higher Education and Research, Belagavi, India; Lata Medical Research Foundation, Nagpur, India) and the corresponding U.S. partners (Boston University, Columbia University, Thomas Jefferson University, and RTI International). All women provided informed consent for participation in the study. The study was funded by grants from the U.S. National Institute of Child Health and Human Development.

## Results

From the overall cohort in the India and Pakistan sites from 2012 to 2018, 230,765 women were screened (Fig. 1). Of these women, 229,218 (99.3%) were eligible and consented to take part in the study. Of these pregnant women, 227,541 delivered, 68,590 from the Pakistan site and 158,951 from the two Indian sites. Of these, 49,223 women (6574 Pakistani and 42,649 Indian women) were excluded, because they were not residents of a study cluster (29,539), had a miscarriage (13,531), or had a medically terminated pregnancy (6153). Hence, a total of 178,318 deliveries were eligible for further analysis, 62,016 from the Pakistani site and 116,302 from the two Indian sites. Of these eligible deliveries, Hb data were available for 130,888 women, 18,154 from the Pakistani site and 112,734 from the Indian sites. In both countries, about half of the women were enrolled in the first trimester, about a third in the second trimester and the remainder in the third trimester or after delivery. Only about 1.5% of the women in both sites were enrolled after delivery (data not shown).

To assess whether the women for which we had Hb measurements were similar to those for which Hb measurements were not available, we compared the populations in Supplemental Table S1. While many of the differences were significant, the differences between the two groups were generally small and not likely clinically important.

Table 1 presents the Hb concentrations by country. The mean Hb concentration in the Indian sites was 10.0 g/dL (SD 1.0) and 9.5 g/dL (SD 1.7) in the Pakistani site. 6.9% of the pregnant Pakistani women and 0.2% of the Indian women had Hb concentrations of < 7.0 g/dL. 10.5% of Pakistani women had Hb concentrations from 7.0–7.9 g/dL and 0.9% of Indian women were in this category. 18.6% of Pakistani women had Hb concentrations between 8.0–8.9 g/dL, while 7.1% of the Indian women had Hb concentrations in this category. Thus, we observed substantial differences in the percent of very low Hb values between women at the Pakistani and Indian sites. On the other hand, 20.5% of Pakistani women, compared to 38.6% of Indian women, had Hb concentrations of 10.0–10.9 g/dL. 16.8% of Pakistani women and 14.3% of Indian women had Hb concentrations of 11.0–12.9 g/dL. Very few women had Hb concentrations of ≥13.0 g/dL in the Pakistani site (1.9%) and in the Indian sites (1.1%).

**Table 1:** Hemoglobin concentrations among pregnant women in Indian and Pakistan Global Network study sites, 2012–2018

	Total	Pakistan	India
Deliveries, N	130,888	18,154	112,734
Hemoglobin g/dL, N (%)			
Very low (< 7.0)	1523 (1.2)	1255 (6.9)	268 (0.2)
Low (7.0–7.9)	2903 (2.2)	1911(10.5)	992 (0.9)
Low (8.0–8.9)	11,416 (8.7)	3379 (18.6)	8037 (7.1)
Low (9.0–9.9)	46,946 (35.9)	4486 (24.7)	42,460 (37.7)
Low normal (10.0–10.9)	47,245 (36.1)	3715 (20.5)	43,530 (38.6)
Normal (11.0–12.9)	19,217 (14.7)	3054 (16.8)	16,163 (14.3)
High (≥ 13.0)	1638 (1.3)	354 (1.9)	1284 (1.1)
Hemoglobin mean (SD^a^)	9.9 (1.1)	9.5 (1.7)	10.0 (1.0)

^a^*SD* standard deviation

Tables 2 and 3 describe Hb concentrations by the socio-demographic and clinical characteristics of pregnant women for the Pakistani and Indian sites. In both sites, the percent of women < 20 years of age was low, 4.2% in Pakistan and 6.7% in India. Women with a primary or higher level of education were much more common in the Indian site, 90.9% vs 17.1% in the Pakistani site. The percent of women with parity > 4 was higher in Pakistan 31.2% vs 1.3% in India. Women enrolled in both countries tended to have a body mass index (BMI) < 18.5 kg/m^2^, 28.7% in Pakistan, and 37.0% in India.

**Table 2:** Socio Demographic and Clinical Characteristics of Pregnant Women by Hemoglobin Concentration for Pakistan

Maternal Characteristics	Overall^1^ N (%)	Maternal Hemoglobin g/dL^1^, N (%)							*P*-value^2^
		Very low(< 7.0)	Low(7.0–7.9)	Low(8.0–8.9)	Low(9.0–9.9)	Low normal(10.0–10.9)	Normal(11.0–12.9)	High(≥ 13.0)	
Deliveries, N	18,154	1255 (6.9)	1911 (10.5)	3379 (18.6)	4486 (24.7)	3715 (20.5)	3054 (16.8)	354 (1.9)	
Maternal age									<.0001
< 20	757 (4.2)	19 (2.5)	79 (10.4)	108 (14.3)	161 (21.3)	171 (22.6)	195 (25.8)	24 (3.2)	
20–35	16,240 (89.5)	1115 (6.9)	1669 (10.3)	3004 (18.5)	4003 (24.6)	3356 (20.7)	2774 (17.1)	319 (2.0)	
> 35	1157 (6.4)	121 (10.5)	163 (14.1)	267 (23.1)	322 (27.8)	188 (16.2)	85 (7.3)	11 (1.0)	
Education									<.0001
No formal education	15,042 (82.9)	1160 (7.7)	1716 (11.4)	2941 (19.6)	3775 (25.1)	2972 (19.8)	2214 (14.7)	264 (1.8)	
Primary/Secondary	2838 (15.6)	93 (3.3)	184 (6.5)	410 (14.4)	650 (22.9)	670 (23.6)	748 (26.4)	83 (2.9)	
University+	274 (1.5)	2 (0.7)	11 (4.0)	28 (10.2)	61 (22.3)	73 (26.6)	92 (33.6)	7 (2.6)	
Parity									<.0001
0	3766 (21.0)	135 (3.6)	333 (8.8)	545 (14.5)	813 (21.6)	887 (23.6)	944 (25.1)	109 (2.9)	
1–3	8551 (47.8)	569 (6.7)	833 (9.7)	1586 (18.5)	2274 (26.6)	1766 (20.7)	1362 (15.9)	161 (1.9)	
≥ 4	5588 (31.2)	531 (9.5)	725 (13.0)	1221 (21.9)	1347 (24.1)	1001 (17.9)	687 (12.3)	76 (1.4)	
BMI									<.0001
< 18.5	5206 (28.7)	390 (7.5)	577 (11.1)	987 (19.0)	1215 (23.3)	1031 (19.8)	889 (17.1)	117 (2.2)	
18.5–25	10,794 (59.5)	769 (7.1)	1185 (11.0)	2085 (19.3)	2735 (25.3)	2161 (20.0)	1685 (15.6)	174 (1.6)	
≥ 25	2140 (11.8)	95 (4.4)	147 (6.9)	306 (14.3)	528 (24.7)	523 (24.4)	478 (22.3)	63 (2.9)	
Multiple pregnancy									0.3639
Yes	194 (1.1)	13 (6.7)	24 (12.4)	38 (19.6)	44 (22.7)	40 (20.6)	30 (15.5)	5 (2.6)	
No	17,906 (98.9)	1231 (6.9)	1881 (10.5)	3335 (18.6)	4433 (24.8)	3668 (20.5)	3014 (16.8)	344 (1.9)	
Gestational age at enrollment									<.0001
1st trimester (< 13,0)	8015 (45.2)	371 (4.6)	675 (8.4)	1286 (16.0)	1817 (22.7)	1758 (21.9)	1875 (23.4)	233 (2.9)	
2nd trimester (13,0-23,6)	5547 (31.3)	434 (7.8)	616 (11.1)	1071 (19.3)	1393 (25.1)	1163 (21.0)	786 (14.2)	84 (1.5)	
3rd trimester/after delivery (≥ 24,0)	4166 (23.5)	382 (9.2)	557 (13.4)	938 (22.5)	1178 (28.3)	719 (17.3)	358 (8.6)	34 (0.8)	
Inter-delivery interval, N (%)									0.3340
≤ 12 months	569 (4.1)	51 (9.0)	70 (12.3)	101 (17.8)	161 (28.3)	93 (16.3)	79 (13.9)	14 (2.5)	
12–24 months	5263 (37.6)	393 (7.5)	561 (10.7)	1083 (20.6)	1316 (25.0)	1068 (20.3)	765 (14.5)	77 (1.5)	
> 24 months	8169 (58.3)	639 (7.8)	913 (11.2)	1606 (19.7)	2116 (25.9)	1570 (19.2)	1181 (14.5)	144 (1.8)	

^1^The overall column displays % of each characteristic, while the hemoglobin columns display % of each hemoglobin category within each maternal characteristic subgroup

^2^*P*-values based on a Cochran-Mantel-Haenszel test for row mean differences based on standard mid-rank (modridit) scores stratified by cluster

**Table 3:** Socio Demographic and Clinical Characteristics of Pregnant Women by Hemoglobin Concentration for India

Maternal Characteristics	Overall^1^ N (%)	Maternal Hemoglobin g/dL^1^, N (%)							*P*-value^2^
		Very low(< 7.0)	Low(7.0–7.9)	Low(8.0–8.9)	Low(9.0–9.9)	Low normal(10.0–10.9)	Normal(11.0–12.9)	High(≥ 13.0)	
Deliveries, N	112,734	268 (0.2)	992 (0.9)	8037 (7.1)	42,460 (37.7)	43,530 (38.6)	16,163 (14.3)	1284 (1.1)	
Maternal age, N (%)									<.0001
< 20	7496 (6.7)	31 (0.4)	81 (1.1)	488 (6.5)	2470 (33.0)	2684 (35.8)	1522 (20.3)	220 (2.9)	
20–35	104,760 (93.0)	236 (0.2)	908 (0.9)	7504 (7.2)	39,835 (38.0)	40,651 (38.8)	14,565 (13.9)	1061 (1.0)	
> 35	446 (0.4)	1 (0.2)	3 (0.7)	41 (9.2)	138 (30.9)	186 (41.7)	74 (16.6)	3 (0.7)	
Education, N (%)									<.0001
No formal education	10,249 (9.1)	67 (0.7)	143 (1.4)	1005 (9.8)	4165 (40.6)	3582 (34.9)	1184 (11.6)	103 (1.0)	
Primary/Secondary	86,809 (77.1)	175 (0.2)	761 (0.9)	6295 (7.3)	33,485 (38.6)	33,306 (38.4)	11,834 (13.6)	953 (1.1)	
University+	15,551 (13.8)	26 (0.2)	84 (0.5)	731 (4.7)	4766 (30.6)	6586 (42.4)	3130 (20.1)	228 (1.5)	
Parity, N (%)									<.0001
0	49,929 (44.4)	124 (0.2)	397 (0.8)	3090 (6.2)	17,386 (34.8)	19,867 (39.8)	8332 (16.7)	733 (1.5)	
1–3	61,118 (54.3)	138 (0.2)	561 (0.9)	4734 (7.7)	24,366 (39.9)	23,133 (37.8)	7647 (12.5)	539 (0.9)	
≥ 4	1432 (1.3)	6 (0.4)	31 (2.2)	206 (14.4)	598 (41.8)	424 (29.6)	157 (11.0)	10 (0.7)	
BMI, N (%)									<.0001
< 18.5	41,555 (37.0)	114 (0.3)	427 (1.0)	3854 (9.3)	17,408 (41.9)	14,622 (35.2)	4746 (11.4)	384 (0.9)	
18.5–25	64,980 (57.9)	147 (0.2)	532 (0.8)	3932 (6.1)	23,519 (36.2)	26,312 (40.5)	9809 (15.1)	729 (1.1)	
≥ 25	5753 (5.1)	6 (0.1)	26 (0.5)	223 (3.9)	1358 (23.6)	2397 (41.7)	1573 (27.3)	170 (3.0)	
Multiple pregnancy, N (%)									0.2983
Yes	991 (0.9)	1 (0.1)	14 (1.4)	77 (7.8)	382 (38.5)	356 (35.9)	152 (15.3)	9 (0.9)	
No	111,596 (99.1)	266 (0.2)	978 (0.9)	7943 (7.1)	42,022 (37.7)	43,116 (38.6)	15,998 (14.3)	1273 (1.1)	
Gestational age at enrollment, N (%)									<.0001
1st trimester (< 13,0)	59,855 (53.3)	133 (0.2)	494 (0.8)	4305 (7.2)	22,355 (37.3)	21,711 (36.3)	9771 (16.3)	1086 (1.8)	
2nd trimester (13,0-23,6)	38,536 (34.3)	94 (0.2)	366 (0.9)	2794 (7.3)	15,180 (39.4)	15,504 (40.2)	4458 (11.6)	140 (0.4)	
3rd trimester/after delivery (≥ 24,0)	13,953 (12.4)	39 (0.3)	127 (0.9)	914 (6.6)	4795 (34.4)	6168 (44.2)	1863 (13.4)	47 (0.3)	
Inter-delivery interval, N (%)									<.0001
≤ 12 months	903 (2.4)	4 (0.4)	15 (1.7)	76 (8.4)	347 (38.4)	330 (36.5)	122 (13.5)	9 (1.0)	
12–24 months	10,799 (28.9)	20 (0.2)	109 (1.0)	893 (8.3)	4339 (40.2)	3725 (34.5)	1563 (14.5)	150 (1.4)	
> 24 months	25,662 (68.7)	75 (0.3)	252 (1.0)	1857 (7.2)	9461 (36.9)	9747 (38.0)	3938 (15.3)	332 (1.3)	

^1^The overall column displays % of each characteristic, while the hemoglobin columns display % of each hemoglobin category within each maternal characteristic subgroup

^2^*P*-values based on a Cochran-Mantel-Haenszel test for row mean differences based on standard mid-rank (modridit) scores stratified by cluster

In both the Pakistani and Indian sites, younger women and primiparous women were more likely to have normal or higher Hb concentrations, while women of higher parity were more likely to have lower Hb concentrations. In the Pakistani site, women > 35 were also more likely to have lower Hb concentrations. Higher educated women in both sites tended to have normal Hb concentrations, while women with no formal education were more likely to have low Hb concentrations. In all of the sites, women with a BMI ≥ 25 kg/m^2^ tended towards higher Hb concentrations, however in India only women BMI < 18.5 kg/m^2^ tended to have lower Hb concentrations of 8.0–9.9 g/dL. Multiple pregnancy was not statistically associated with Hb concentrations. In the Pakistani site, inter-delivery interval was not associated with Hb level. However, in the Indian sites, longer inter-delivery intervals tended to be associated with a greater proportion of normal Hb concentrations. Gestational age at enrollment (and a proxy for the gestational age at which the Hb measurement was done) was associated with the Hb concentrations. In the Pakistani site, a higher proportion of women who enrolled late had lower Hb concentrations.

Tables 4 and 5 display the unadjusted rates of adverse maternal and fetal/neonatal outcomes overall and by Hb category. Maternal and neonatal mortality and stillbirth as well as the other adverse outcomes including antepartum and postpartum hemorrhage, PTB, and LBW were observed more often in the Pakistani site than in the Indian sites. Hypertensive disease/pre-eclampsia/eclampsia, obstructed labor and cesarean delivery were observed more often in the Indian sites than in the Pakistani site.

**Table 4:** Maternal and Fetal/Neonatal Outcomes by Hemoglobin Concentration for Pakistan

	Overall	Maternal Hemoglobin g/dL						
		Very low(< 7.0)	Low(7.0–7.9)	Low(8.0–8.9)	Low(9.0–9.9)	Low normal(10.0–10.9)	Normal(11.0–12.9)	High(≥ 13.0)
**Maternal Outcomes**								
Deliveries, N	18,154	1255	1911	3379	4486	3715	3054	354
Maternal death < 42 days(Rate/100,000 deliveries)	65 (359)	8 (641)	10 (524)	16 (475)	8 (179)	11 (297)	9 (296)	3 (847)
Antepartum hemorrhage, N (%)	371 (2.0)	44 (3.5)	34 (1.8)	74 (2.2)	80 (1.8)	77 (2.1)	54 (1.8)	8 (2.3)
Postpartum hemorrhage, N (%)	479 (2.6)	47 (3.8)	64 (3.4)	89 (2.6)	116 (2.6)	93 (2.5)	57 (1.9)	13 (3.7)
Hypertensive disease/pre- eclampsia/eclampsia, N (%)	481 (2.7)	34 (2.7)	44 (2.3)	82 (2.4)	114 (2.5)	95 (2.6)	99 (3.2)	13 (3.7)
Obstructed labor, N (%)	1275 (7.0)	87 (6.9)	124 (6.5)	215 (6.4)	269 (6.0)	274 (7.4)	271 (8.9)	35 (9.9)
C-delivery, N (%)	2713 (15.0)	104 (8.3)	173 (9.1)	390 (11.5)	644 (14.4)	649 (17.5)	670 (22.0)	83 (23.5)
Preterm, N (%)	4670 (25.8)	405 (32.3)	536 (28.2)	883 (26.2)	1161 (25.9)	933 (25.1)	669 (21.9)	83 (23.5)
**Fetal/Neonatal Outcomes**								
Births, N	18,354	1269	1936	3420	4531	3755	3084	359
Stillbirth, N (Rate/1000)	889 (48.5)	105 (82.9)	115 (59.6)	163 (47.7)	208 (45.9)	162 (43.2)	118 (38.3)	18 (50.3)
Neonatal mortality < 7 days, N (Rate/1000)	709 (40.7)	61 (52.8)	79 (43.6)	131 (40.3)	183 (42.4)	121 (33.8)	113 (38.2)	21 (61.8)
Neonatal mortality < 28 days, N (Rate/1000)	889 (51.1)	80 (69.2)	101 (55.7)	169 (52.0)	222 (51.5)	157 (43.8)	137 (46.4)	23 (67.6)
Birth weight (g)^a^, Mean (SD)	2702 (491)	2587 (523)	2672 (491)	2687 (493)	2702 (488)	2727 (476)	2754 (482)	2705 (545)
Low birth weight (< 2500 g)^a^, N (%)	3950 (22.2)	367 (30.7)	455 (24.6)	772 (23.2)	929 (21.1)	752 (20.6)	586 (19.6)	89 (25.9)

^a^Birth weight measured within 7 days of delivery

**Table 5:** Maternal and Fetal/Neonatal Outcomes by Hemoglobin Concentration for India

	Overall	Maternal Hemoglobin g/dL						
		Very low(< 7.0)	Low(7.0–7.9)	Low(8.0–8.9)	Low(9.0–9.9)	Low normal(10.0–10.9)	Normal(11.0–12.9)	High(≥ 13.0)
**Maternal Outcomes**								
Deliveries, N	112,734	268	992	8037	42,460	43,530	16,163	1284
Maternal death < 42 days (Rate/100,000 deliveries)	134 (119)	0 (0)	1 (101)	18 (224)	41 (97)	47 (108)	23 (142)	4 (312)
Antepartum hemorrhage, N (%)	739 (0.7)	6 (2.2)	11 (1.1)	60 (0.7)	301 (0.7)	239 (0.5)	114 (0.7)	8 (0.6)
Postpartum hemorrhage, N (%)	679 (0.6)	10 (3.8)	14 (1.4)	70 (0.9)	209 (0.5)	233 (0.5)	131 (0.8)	12 (1.0)
Hypertensive disease/pre- eclampsia/eclampsia, N (%)	3685 (3.3)	18 (6.7)	38 (3.8)	255 (3.2)	1287 (3.0)	1314 (3.0)	675 (4.2)	98 (7.6)
Obstructed labor, N (%)	11,546 (10.3)	27 (10.1)	90 (9.1)	749 (9.3)	3970 (9.4)	4557 (10.5)	1982 (12.3)	171 (13.3)
C-delivery, N (%)	28,706 (25.5)	64 (23.9)	203 (20.5)	1766 (22.0)	9664 (22.8)	11,412 (26.2)	5172 (32.0)	425 (33.1)
Preterm, N (%)	12,740 (11.3)	43 (16.2)	141 (14.2)	1050 (13.1)	5060 (12.0)	4629 (10.7)	1676 (10.4)	141 (11.0)
**Fetal/Neonatal Outcomes**								
Births, N	113,749	269	1006	8116	42,853	43,893	16,319	1293
Stillbirth, N (Rate/1000)	2926 (25.7)	15 (55.8)	36 (35.8)	245 (30.2)	1166 (27.2)	1038 (23.7)	392 (24.0)	34 (26.3)
Neonatal mortality < 7 days, N (Rate/1000)	2097 (18.9)	10 (39.4)	30 (31.0)	182 (23.1)	856 (20.6)	732 (17.1)	266 (16.7)	21 (16.7)
Neonatal mortality < 28 days, N (Rate/1000)	2593 (23.4)	12 (47.2)	33 (34.1)	228 (29.0)	1038 (24.9)	926 (21.6)	328 (20.6)	28 (22.3)
Birth weight (g)^a^, Mean (SD)	2707 (462)	2641 (505)	2644 (481)	2670 (472)	2687 (466)	2720 (450)	2744 (469)	2728 (462)
Low birth weight (< 2500 g)^a^, N (%)	21,575 (19.2)	69 (26.6)	253 (25.7)	1783 (22.3)	8436 (20.0)	7787 (18.0)	2969 (18.5)	278 (21.9)

^a^Birth weight measured within 7 days of delivery

To determine the association of the Hb concentrations and the adverse outcomes, stratified by the Pakistani (Table 6) and the Indian sites (Table 7), we compared the risk of the adverse outcomes for each Hb category to the risk of those outcomes in women with a Hb concentration of 11.0–12.9 g/dL, our reference group. The risks of outcomes were adjusted for potential confounders including maternal age, education level, parity, BMI and gestational age at enrollment. Because maternal deaths were relatively rare, the models did not converge to produce estimates of risk for maternal mortality by Hb concentration for either site. For the Pakistani site, women with Hb concentrations < 7 g/dL had a higher risk of antepartum hemorrhage compared to women with normal Hb concentrations; for post-partum hemorrhage, women with Hb concentrations < 7.9 g/dL showed higher risk. Hb concentrations were not associated with hypertensive disease or obstructed labor. Compared to women with normal Hb concentrations, women with Hb concentrations < 11 g/dL were less likely to have a cesarean delivery and had a higher risk for PTB. Stillbirths were generally more common in the groups with a Hb concentration < 10 g/dL, with 3 of the 4 < 10 g/dL Hb concentration groups having a statistically greater risk of stillbirth than the group with a Hb concentration of 11–12.9 g/dL. Neonatal mortality, whether at < 7 days or < 28 days, was higher in the groups with Hb concentrations < 7 g/dL and in the women with a Hb concentration of > 13 g/dL, a U-shaped relationship. Birthweight was lower in all the groups with a Hb concentration < 11 g/dL compared to the reference group. LBW also had a U-shaped relationship with higher risk for Hb concentrations above and below 11.0 to 12.9 g/dL.

**Table 6:** Adjusted Risk of Maternal and Fetal/Neonatal Outcomes by Hemoglobin Concentration for Pakistan

Outcomes	< 7.0 g/dL vs.11.0–12.9 g/dL		7.0–7.9 g/dL vs.11.0–12.9 g/dL		8.0–8.9 g/dL vs.11.0–12.9 g/dL		9.0–9.9 g/dL vs.11.0–12.9 g/dL		10.0–10.9 g/dL vs.11.0–12.9 g/dL		≥ 13.0 g/dL vs.11.0–12.9 g/dL	
	RR/MD(95% CI)	*P*-value	RR/MD(95% CI)	*P*-value	RR/MD(95% CI)	*P*-value	RR/MD(95% CI)	*P*-value	RR/MD(95% CI)	*P*-value	RR/MD(95% CI)	*P*-value
**Maternal Outcomes**												
Maternal death < 42 days	–	–	–	–	–	–	–	–	–	–	–	–
Antepartum hemorrhage	1.86(1.34, 2.57)	0.0002	0.86(0.55, 1.37)	0.5356	1.19(0.84, 1.68)	0.3400	1.09(0.80, 1.47)	0.5843	1.20(0.80, 1.80)	0.3873	1.37(0.80, 2.36)	0.2544
Postpartum hemorrhage	1.68(1.16, 2.43)	0.0064	1.48(1.02, 2.15)	0.0403	1.22(0.81, 1.85)	0.3381	1.29(0.86, 1.95)	0.2196	1.22(0.86, 1.75)	0.2703	1.61(0.84, 3.07)	0.1478
Hypertensive disease /pre-eclampsia/ eclampsia^2^	1.18(0.81, 1.73)	0.3910	0.98(0.63, 1.51)	0.9137	0.99(0.76, 1.29)	0.9573	0.95(0.78, 1.16)	0.5973	0.92(0.69, 1.22)	0.5554	0.99(0.59, 1.65)	0.9650
Obstructed labor	1.13(0.89, 1.45)	0.3166	0.98(0.82, 1.18)	0.8669	1.00(0.85, 1.18)	0.9948	0.92(0.80, 1.06)	0.2600	1.00(0.88, 1.13)	0.9970	1.02(0.66, 1.57)	0.9288
Cesarean delivery	0.64(0.53, 0.76)	<.0001	0.67(0.55, 0.82)	0.0001	0.77(0.69, 0.86)	<.0001	0.86(0.79, 0.94)	0.0006	0.94(0.89, 0.99)	0.0292	0.98(0.82, 1.16)	0.7896
Preterm	1.63(1.42, 1.87)	<.0001	1.35(1.21, 1.51)	<.0001	1.26(1.17, 1.35)	<.0001	1.24(1.13, 1.35)	<.0001	1.17(1.06, 1.29)	0.0013	1.07(0.88, 1.31)	0.5043
**Fetal/Neonatal Outcomes**												
Stillbirth	2.25(1.76, 2.88)	<.0001	1.55(1.17, 2.06)	0.0021	1.22(0.94, 1.59)	0.1408	1.22(1.05, 1.42)	0.0115	1.15(0.93, 1.43)	0.2022	1.27(0.84, 1.90)	0.2544
Neonatal mortality < 7 days	1.42(1.05, 1.93)	0.0249	1.11(0.83, 1.50)	0.4808	1.12(0.88, 1.43)	0.3648	1.13(0.95, 1.34)	0.1675	0.88(0.71, 1.09)	0.2277	1.64(1.07, 2.50)	0.0225
Neonatal mortality < 28 days	1.56(1.13, 2.15)	0.0071	1.20(0.92, 1.56)	0.1786	1.19(0.95, 1.49)	0.1371	1.14(0.97, 1.35)	0.1140	0.96(0.77, 1.18)	0.6769	1.49(1.00, 2.23)	0.0495
Birth weight (g)	−177.8(− 212.0, − 143.7)	<.0001	−86.1(−115.3, −56.9)	<.0001	−74.6(−99.4, −49.7)	<.0001	−64.2(−87.5, −41.0)	<.0001	−33.4(−57.3, − 9.6)	0.0060	−43.9(−98.5, 10.8)	0.1157
Low birth weight(< 2500 g)	1.65(1.49, 1.82)	<.0001	1.31(1.19, 1.44)	<.0001	1.23(1.14, 1.33)	<.0001	1.16(1.06, 1.27)	0.0018	1.08(1.00, 1.18)	0.0613	1.29(1.06, 1.56)	0.0108

^1^Relative risks (RR) and *p-*values for binary outcomes are obtained from generalized linear models with a binomial distribution assumption and log-link accounting for potential confounders of age, education, parity, BMI and GA at enrollment controlling for within-cluster correlation. For the continuous outcome of birthweight, the mean differences (MD) and *p*-values are from a linear mixed model controlling for cluster as a random effect and accounting for age, education, parity, BMI and GA at enrollment

^2^Generalized linear model with a Poisson distribution assumption and log-link

**Table 7:** Adjusted Risk of Maternal and Fetal/Neonatal Outcomes by Hemoglobin Concentration for India

Outcomes	< 7.0 g/dL vs.11.0–12.9 g/dL		7.0–7.9 g/dL vs.11.0–12.9 g/dL		8.0–8.9 g/dL vs.11.0–12.9 g/dL		9.0–9.9 g/dL vs.11.0–12.9 g/dL		10.0–10.9 g/dL vs.11.0–12.9 g/dL		≥ 13.0 g/dL vs.11.0–12.9 g/dL	
	RR/MD(95% CI)	*P*-value	RR/MD(95% CI)	*P*-value	RR/MD(95% CI)	*P*-value	RR/MD(95% CI)	*P*-value	RR/MD(95% CI)	*P*-value	RR/MD(95% CI)	*P*-value
**Maternal Outcomes**												
Maternal death < 42 days	–	–	–	–	–	–	–	–	–	–	–	–
Antepartum hemorrhage	3.28(1.67, 6.45)	0.0006	1.63(0.90, 2.96)	0.1054	1.13(0.74, 1.71)	0.5729	1.17(0.88, 1.57)	0.2802	0.96(0.73, 1.26)	0.7590	0.73(0.27, 1.97)	0.5381
Postpartum hemorrhage^2^	4.48(2.62, 7.66)	<.0001	1.74(0.95, 3.17)	0.0726	1.33(0.89, 1.98)	0.1606	0.82(0.63, 1.06)	0.1294	0.88(0.72, 1.09)	0.2524	0.82(0.28, 2.42)	0.7166
Hypertensive disease /pre-eclampsia/ eclampsia^2^	1.89(1.12, 3.18)	0.0169	1.07(0.73, 1.59)	0.7247	1.04(0.94, 1.16)	0.4312	0.93(0.84, 1.02)	0.1308	0.87(0.79, 0.95)	0.0024	1.46(1.13, 1.89)	0.0034
Obstructed labor	0.96(0.71, 1.31)	0.7919	0.86(0.70, 1.06)	0.1593	0.84(0.75, 0.94)	0.0019	0.86(0.81, 0.92)	<.0001	0.92(0.88, 0.96)	0.0004	1.05(0.86, 1.28)	0.6263
C-delivery^2^	0.96(0.80, 1.15)	0.6382	0.76(0.68, 0.85)	<.0001	0.80(0.76, 0.85)	<.0001	0.81(0.78, 0.84)	<.0001	0.87(0.85, 0.89)	<.0001	1.01(0.93, 1.10)	0.7884
Preterm	1.47(1.19, 1.82)	0.0004	1.35(1.16, 1.58)	0.0001	1.23(1.15, 1.32)	<.0001	1.14(1.08, 1.21)	<.0001	1.04(0.98, 1.10)	0.1751	0.99(0.87, 1.14)	0.9372
**Fetal/Neonatal Outcomes**												
Stillbirth	2.20(1.35, 3.60)	0.0017	1.45(0.94, 2.22)	0.0931	1.26(1.10, 1.45)	0.0011	1.14(1.02, 1.28)	0.0246	1.00(0.90, 1.11)	0.9965	1.03(0.72, 1.47)	0.8790
Neonatal mortality < 7 days	2.30(1.36, 3.91)	0.0019	1.84(1.27, 2.67)	0.0012	1.44(1.15, 1.81)	0.0013	1.29(1.12, 1.50)	0.0006	1.08(0.93, 1.26)	0.3240	0.93(0.60, 1.44)	0.7547
Neonatal mortality < 28 days	2.02(1.19, 3.43)	0.0094	1.63(1.13, 2.36)	0.0090	1.44(1.17, 1.78)	0.0006	1.25(1.08, 1.46)	0.0034	1.10(0.95, 1.27)	0.2197	1.02(0.70, 1.46)	0.9322
Birth weight (g)	−92.4(−148.7, −36.1)	0.0013	−86.2(−115.9, −56.5)	<.0001	−55.5(−68.0, −43.1)	<.0001	−38.9(−47.4, −30.5)	<.0001	−9.8(− 18.1, − 1.4)	0.0226	−26.1(−52.4, 0.1)	0.0509
Low birth weight(< 2500 g)	1.32(1.05, 1.66)	0.0171	1.33(1.18, 1.51)	<.0001	1.16(1.10, 1.22)	<.0001	1.06(1.00, 1.12)	0.0524	0.99(0.95, 1.03)	0.5591	1.08(0.95, 1.24)	0.2390

^1^Relative risks (RR) and *p-*values for binary outcomes are obtained from generalized linear models with a binomial distribution assumption and log-link accounting for potential confounders of age, education, parity, BMI and GA at enrollment controlling for within-cluster correlation. For the continuous outcome of birthweight, the mean differences (MD) and *p-*values are from a linear mixed model controlling for cluster as a random effect and accounting for age, education, parity, BMI and GA at enrollment

^2^Generalized linear model with a Poisson distribution assumption and log-link

**Fig. 2:**
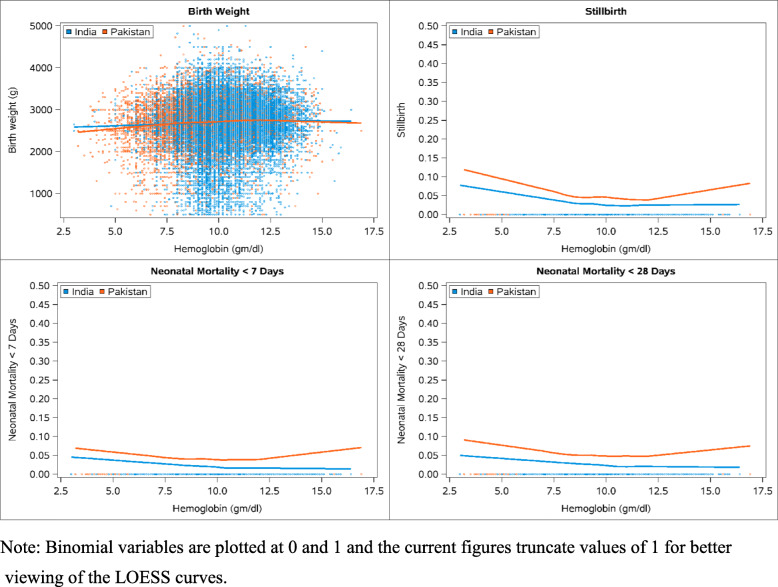
LOESS Plots of Fetal/Neonatal Outcomes by Country

In the Indian sites, as in the Pakistani site, both antepartum and postpartum hemorrhage were associated with very low (< 7.0 g/dL) Hb concentrations. Hypertensive disease was more common in women with very low and very high Hb concentrations, suggesting a U-shaped relationship. Furthermore, women with a Hb of 10.0–10.9 g/dL had lower risk for hypertensive disease compared to 11.0–12.9 g/dL. Risk of obstructed labor and cesarean delivery was lower in the women with Hb values between 8 and 11 g/dL and women with Hb values between 7 and 11 g/dL were also at lower risk for cesarean delivery. Risk of stillbirth was generally higher in women with a Hb < 10 g/dL with the results significant in 3 of the 4 Hb < 10 g/dL categories. Risk of PTB was higher for Indian women with Hb < 10 g/dL. Similar to the Pakistani site, women with a Hb concentration of < 10 g/dL had a higher risk of neonatal mortality, however women with a Hg concentration ≥ 13 g/dL did not show increased risk as in the Pakistani site. In India, women with Hb concentrations < 9.0 g/dL were at higher risk to have babies with LBW and women with Hb concentrations < 11.0 g/dL were more likely to have babies weighing less than those with normal Hb concentrations. The LOESS plots (Fig. 2) illustrate the relationship between Hb concentrations and the outcomes of birthweight, stillbirth and 7 and 28-day mortality for the Indian and Pakistani sites. Figure 2 illustrates that there is a U-shaped relationship between hemoglobin concentrations and outcomes such as neonatal mortality and stillbirth for Pakistan. The relationship between hemoglobin concentrations and birth weight by site showed that both correlations were positive and significantly different from zero, but not high (Pakistan: R^2^ = 0.073, *p* < 0.0001 and India: R^2^ = 0.049, *p <* 0.0001).

## Discussion

This study had several important findings. First, although India and Pakistan originally were part of the same country and separated only about 70 years ago, the populations we studied are quite different in a number of demographic characteristics. The Pakistani population studied was far less educated and much more likely to have a higher parity than the Indian population. Both populations had high numbers of women with a low BMI although the Pakistani population had twice the rate of obese women (BMI > 25 kg/m^2^). Low Hb concentrations were common in both populations although the proportion of women with very low Hb concentrations was much higher in Pakistan.

The relationships between maternal Hb concentrations and adverse outcomes were generally similar between the Pakistani and India sites, although there were differences. Antepartum and post-partum hemorrhage and stillbirth were associated with low Hb concentrations in enrolled women in both countries. Cesarean delivery also occurred less frequently at the lower Hb concentrations for both sites. Lower Hb concentrations were also associated with lower risk of obstructed labor in India. Hypertensive disease was not associated with Hb concentrations in the Pakistani site but was associated with a U-shaped relationship with Hb concentrations in India. In both populations, LBW was more common and low mean birthweight were always more common at the lowest Hb concentrations. In the Pakistani site, LBW tended to occur more often at the highest Hb concentrations as well. Seven and 28-day neonatal mortality was more common at the lower Hb concentrations, and in the Pakistani site, there was a U-shaped relationship with both high and low Hb.

There are several mechanisms through which low and high Hb concentrations may be associated with adverse fetal, neonatal, and maternal outcomes. For example, the underlying cause of low Hb concentrations is most often due to iron or vitamin deficiency resulting in impaired transport of oxygen to the uterus, placenta, and fetus. This mechanism might explain the increase in preterm birth, low birth weight and perinatal deaths associated with low Hb [20]. On the other hand, the association of high Hb concentrations with adverse outcomes might be due to other mechanisms. Higher Hb concentrations may not be a marker for increased red cell production, but instead may be a result of failure for the plasma volume to expand. The literature suggests that failure of the Hb concentrations to fall is associated with up to a threefold increased risk of pre-eclampsia and the birth of small for gestational age infants and preterm delivery [21–24]. Thus, an elevated Hb level is an indicator for possible pregnancy complications associated with poor plasma volume expansion, and should not be mistaken for good iron status [16].

Furthermore, it has been suggested that high Hb concentrations may restrict intrauterine growth as a consequence of high blood viscosity [14]. The increased viscosity related to low uterine arterial blood flow results in reduced oxygen delivery to the fetus. Thus, extremes of Hb concentrations during pregnancy appear to be associated with an increase in adverse outcomes. This issue may be an important consideration for setting standards for appropriate Hb concentrations during pregnancy. This U-shaped relationship of Hb concentrations has been found with adverse outcomes such as stillbirth, preterm birth, and LBW in more developed countries [11, 16]. Hence, through the present study, we were able to confirm evidence of a U-shaped relationship between Hb concentration and several adverse pregnancy outcomes among South Asian pregnant women.

### Strengths and limitations

One of the biggest strengths of our study is the size of the cohort. To our knowledge, this is the largest cohort study that looked specifically at the extremes of Hb concentrations and its association with maternal and neonatal outcomes in South Asian populations in India and Pakistan. Despite these strengths, our study had some limitations. First, only 29% of the Pakistani women had a Hb measured. There were also some differences in the women with Hb measurements and those without, and although these differences were small, the results may not be generalizable to all women in the study. Also, we did not record the specific gestational age in pregnancy when the Hb concentrations were measured, although at all sites the Hb level is taken at the first antenatal visit. Because Hb concentrations fell slightly as the pregnancy advanced, we adjusted for the gestational age of the first visit to reduce confounding. Another limitation of our study was that our gestational age data and the rates of PTB were generally based on LMP data rather than on a gestational age determined by an early ultrasound. Although there could be bias among those with Hb data available, we are not aware of any systematic differences in Hb collection that may have biased the relationships we report.

## Conclusions

Based on our findings, both low and high values of Hb should be considered risk factors for the mother and fetus. Further research is required to understand the biological processes that underlie our results. In addition, studies are recommended to identify whether the Pakistani women have very low Hb concentrations due to dietary causes (iron and folic acid deficiencies), paracytic infections, or whether there are some underlying hereditary disorders that might be prevalent among the women such as red cell abnormalities or hemoglobinopathies.

## Supplementary information

**Additional file 1: Supplementary Table S1:** Socio Demographic and Clinical Characteristics of Pregnant Women by Hemoglobin Availability (DOCX 25 kb)

## Data Availability

Data from the study will be available at the NICHD data repository (N-DASH): https://dash.nichd.nih.gov/

## Abbreviations

ANC: Antenatal Care
BMI: Body Mass Index
LBW: Low birth weight
LMIC: Low-middle income countries
MNHR: Maternal Newborn Health Registry
PTB: Preterm birth
RA: Registry Administrator
