# Supplementary material for: Hemoglobin concentrations and adverse birth outcomes in South Asian pregnant women: findings from a prospective Maternal and Neonatal Health Registry
Source: Reprod Health. 2020 Nov 30;17(Suppl 2):154. doi: 10.1186/s12978-020-01006-6 (PMC7706196; doi:10.1186/s12978-020-01006-6)
Supplement: Supplementary file 1 — Additional file 1: Supplementary Table S1: Socio Demographic and Clinical Characteristics of Pregnant Women by Hemoglobin Availability (DOCX 25 kb) [file 12978_2020_1006_MOESM1_ESM.docx]

| **Supplementary Table S1: Socio Demographic and Clinical Characteristics of Pregnant Women by Hemoglobin Availability** |
| --- |

|  | Pakistan | | | | India | | | |
| --- | --- | --- | --- | --- | --- | --- | --- | --- |
| Maternal Characteristics | Overall | Hemoglobin Measured | Hemoglobin Not Measured | p-value^1^ | Overall | Hemoglobin Measured | Hemoglobin Not Measured | p-value^1^ |
| All deliveries | 62,016 | 18,154 | 43,862 |  | 116,302 | 112,734 | 3,568 |  |
| Maternal age |  |  |  | <.0001 |  |  |  | 0.9137 |
| < 20 | 2,598 (4.2) | 757 (4.2) | 1,841 (4.2) |  | 7,941 (6.8) | 7,496 (6.7) | 445 (12.5) |  |
| 20-35 | 55,955 (90.3) | 16,240 (89.5) | 39,715 (90.6) |  | 107,869 (92.8) | 104,760 (93.0) | 3,109 (87.3) |  |
| > 35 | 3,442 (5.6) | 1,157 (6.4) | 2,285 (5.2) |  | 452 (0.4) | 446 (0.4) | 6 (0.2) |  |
| Education |  |  |  | 0.0017 |  |  |  | <.0001 |
| No formal education | 50,884 (82.1) | 15,042 (82.9) | 35,842 (81.8) |  | 10,799 (9.3) | 10,249 (9.1) | 550 (15.5) |  |
| Primary/Secondary | 9,732 (15.7) | 2,838 (15.6) | 6,894 (15.7) |  | 89,405 (77.0) | 86,809 (77.1) | 2,596 (73.1) |  |
| University+ | 1,376 (2.2) | 274 (1.5) | 1,102 (2.5) |  | 15,955 (13.7) | 15,551 (13.8) | 404 (11.4) |  |
| Parity |  |  |  | <.0001 |  |  |  | 0.4957 |
| 0 | 10,813 (18.1) | 3,766 (21.0) | 7,047 (16.8) |  | 51,353 (44.3) | 49,929 (44.4) | 1,424 (40.7) |  |
| 1-3 | 28,096 (47.0) | 8,551 (47.8) | 19,545 (46.6) |  | 63,137 (54.4) | 61,118 (54.3) | 2,019 (57.7) |  |
| ≥ 4 | 20,904 (34.9) | 5,588 (31.2) | 15,316 (36.5) |  | 1,490 (1.3) | 1,432 (1.3) | 58 (1.7) |  |
| BMI |  |  |  | 0.6366 |  |  |  | 0.0004 |
| < 18.5 | 17,390 (28.1) | 5,206 (28.7) | 12,184 (27.9) |  | 42,519 (37.0) | 41,555 (37.0) | 964 (36.0) |  |
| 18.5-25 | 37,144 (60.1) | 10,794 (59.5) | 26,350 (60.3) |  | 66,570 (57.9) | 64,980 (57.9) | 1,590 (59.4) |  |
| ≥ 25 | 7,311 (11.8) | 2,140 (11.8) | 5,171 (11.8) |  | 5,877 (5.1) | 5,753 (5.1) | 124 (4.6) |  |
| Multiple pregnancy |  |  |  | 0.2196 |  |  |  | 0.3672 |
| Yes | 702 (1.1) | 194 (1.1) | 508 (1.2) |  | 1,020 (0.9) | 991 (0.9) | 29 (0.8) |  |
| No | 60,819 (98.9) | 17,906 (98.9) | 42,913 (98.8) |  | 115,131 (99.1) | 111,596 (99.1) | 3,535 (99.2) |  |
| Gestational age at enrollment |  |  |  | <.0001 |  |  |  | <.0001 |
| 1st trimester (< 13,0) | 25,106 (44.9) | 8,015 (45.2) | 17,091 (44.7) |  | 61,464 (53.0) | 59,855 (53.3) | 1,609 (45.2) |  |
| 2nd trimester (13,0-23,6) | 19,291 (34.5) | 5,547 (31.3) | 13,744 (36.0) |  | 40,080 (34.6) | 38,536 (34.3) | 1,544 (43.4) |  |
| 3rd trimester/after delivery (≥ 24,0) | 11,560 (20.7) | 4,166 (23.5) | 7,394 (19.3) |  | 14,358 (12.4) | 13,953 (12.4) | 405 (11.4) |  |
| Inter-delivery interval |  |  |  | <.0001 |  |  |  | 0.3825 |
| ≤ 12 months | 1,466 (5.0) | 569 (4.1) | 897 (5.8) |  | 904 (2.4) | 903 (2.4) | 1 (1.7) |  |
| 12-24 months | 11,215 (38.0) | 5,263 (37.6) | 5,952 (38.3) |  | 10,820 (28.9) | 10,799 (28.9) | 21 (35.6) |  |
| > 24 months | 16,857 (57.1) | 8,169 (58.3) | 8,688 (55.9) |  | 25,699 (68.7) | 25,662 (68.7) | 37 (62.7) |  |

| ^1^ P-values based on a Cochran-Mantel-Haenszel test for row mean differences based on standard mid-rank (modridit) scores stratified by cluster. |
| --- |
